# Differential Cellular and Molecular Effects of Butyrate and Trichostatin A on Vascular Smooth Muscle Cells

**DOI:** 10.3390/ph5090925

**Published:** 2012-09-04

**Authors:** Shirlette G. Milton, Omana P. Mathew, Frank M. Yatsu, Kasturi Ranganna

**Affiliations:** 1 Department of Pharmaceutical Sciences, College of Pharmacy and Health Sciences, Texas Southern University, 3100 Cleburne St, Houston, TX 77004, USA; Email: Milton_SG@tsu.edu (S.G.M.); Mathew_op@tsu.edu (O.P.M.); 2 Department of Neurology, University of Texas Health Science Center at Houston, Houston, TX 77030, USA; Email: fyatsu@att.net (F.M.Y.)

**Keywords:** butyrate, trichostatin A, vascular smooth muscle cells, proliferation, cyclin D1, HDAC

## Abstract

The histone deacetylase (HDAC) inhibitors, butyrate and trichostatin A (TSA), are epigenetic histone modifiers and proliferation inhibitors by downregulating cyclin D1, a positive cell cycle regulator, and upregulating p21Cip1 and INK family of proteins, negative cell cycle regulators. Our recent study indicated cyclin D1 upregulation in vascular smooth muscle cells (VSMC) that are proliferation-arrested by butyrate. Here we investigate whether cyclin D1 upregulation is a unique response of VSMC to butyrate or a general response to HDAC inhibitors (HDACi) by evaluating the effects of butyrate and TSA on VSMC. While butyrate and TSA inhibit VSMC proliferation via cytostatic and cytotoxic effects, respectively, they downregulate cdk4, cdk6, and cdk2, and upregulate cyclin D3, p21Cip1 and p15INK4B, and cause similar effects on key histone H3 posttranslational modifications. Conversely, cyclin D1 is upregulated by butyrate and inhibited by TSA. Assessment of glycogen synthase 3-dependent phosphorylation, subcellular localization and transcription of cyclin D1 indicates that differential effects of butyrate and TSA on cyclin D1 levels are linked to disparity in cyclin D1 gene expression. Disparity in butyrate- and TSA-induced cyclin D1 may influence transcriptional regulation of genes that are associated with changes in cellular morphology/cellular effects that these HDACi confer on VSMC, as a transcriptional modulator.

## 1. Introduction

Atherosclerosis and restenosis, complex pathologies of medium to large blood vessels, are multifactorial vascular proliferative diseases responsible for the global burden of cardiovascular diseases. The majority of cardiovascular diseases are the outcome of vascular remodeling triggered by vascular injury [1,2,3,4,5]. Besides endothelial dysfunction and inflammatory response that is triggered in reaction to injury, VSMC proliferation is also a key etiological factor in the development of atherosclerosis and occlusive neointimal thickening [1,2,3,4,5]. Proliferation of VSMC is also the primary pathophysiological mechanism in different clinical pathologies such as postangioplasty restenosis, in-stent restenosis, vein bypass graft failure and transplant vasculopathy [1,4,6]. Although substantial progress has been made in understanding the etiology and clinical management of vascular proliferative diseases in recent years, they are still life threatening diseases. Therefore, understanding the molecular mechanisms of VSMC proliferation may offer novel insights into disease pathogenesis.

VSMC proliferation, the hallmark of vascular proliferative diseases, involves multiple genes that are transcriptionally regulated in response to injury or mitogenic signals implicating a higher level of regulation exists in VSMC that controls entire gene expression program required for proliferation [7,8]. This regulation involves epigenetic mechanisms, which by altering the chromatin structure and dynamics control transcriptional gene activation that are essential for normal development and maintenance of organisms and to facilitate their interaction with surrounding environment [3,4,9,10,11]. Two of the well-known epigenetic mechanisms, DNA methylation and histone modifications, change the chromatin topography and dynamics that influence gene functions by controlling gene expressions with no changes in their DNA makeup [3,4,9,10,11]. These epigenetic mechanisms are essential for regulation of gene expression by turning parts of the genome “off” and “on” at strategic times at specific sites in response to signals to promote or restrict transcriptional activation via coordination of a set of reversible modifications that causes chromatin remodeling and histone modifications. Currently there is an enormous interest and enthusiasm in linking altered epigenetic mechanisms to human pathologies particularly cancers but it is relatively unexplored area regarding cardiovascular diseases. Dysregulation of epigenetic processes has been linked to changes in many cellular processes including cell growth, cell cycle control, proliferation, differentiation, and cell death by altering the expression and in turn functions of target genes without changing their primary gene structure. Overturning the deregulated epigenetic mechanisms may offer effective treatment strategy for many diseases including cardiovascular disease due to atherosclerosis and restenosis [3,4,9,10,11]. Therefore, understanding the epigenetics of VSMC proliferation and in particular their susceptibility to perturbation by the epigenetic modifiers may offer novel insights into disease pathogenesis and potential epigenetic therapeutic strategies.

The structural modification of histones by acetylation/deacetylation of their *N*-terminal tails is crucial in modulating gene expression, because it affects the accessibility of DNA for the transcription-regulatory protein complexes. The counterbalancing activities of two groups of enzymes, histone acetyl transferases (HATs) and HDACs, contribute to the transcriptional states of chromatin structure. HATs acetylate specific lysine residues of histones, which promote a more relaxed and active chromatin conformation, thus, allowing access of DNA to transcription machinery to turn on gene expression. Conversely, HDACs restore the positive charge on lysine residues by removing acetyl groups that supports condensed chromatin structure resulting in silencing of gene expression by blocking the access of transcription machinery to DNA. Suppression of gene expression by HDACs-mediated epigenetic mechanism has been exploited in the field of cancer to successfully reactivate transcriptionally silent tumor suppressor genes to arrest proliferation of cancer cells and growth [12]. This provides a rationale for targeting HDAC activity inhibition to reactivate transcriptionally silenced genes, which has resulted in an array of both natural and pharmacological HDAC inhibitors (HDACi) [3,11,13,14,15].

Most HDACi exhibit multiple cellular effects, which are linked to chromatin-mediated altered transcriptional activity. In general, they inhibit cell proliferation, stimulate cell differentiation and/or induce apoptosis/cell death by selectively modulating gene expression [13,16,17]. Although relatively limited information is available regarding epigenetic regulation of vascular gene expression, HATs/HDACs appear to regulate expression of some of the genes associated with various cellular processes like inflammation, proliferation, differentiation and matrix modulation [3,18,19,20,21,22,23,24] that are vital to the development of vascular proliferative diseases. Several *in vitro* and *in vivo* studies have been done using HDACi with the intention of targeting VSMC proliferation for the intervention and management of vascular proliferative diseases. However, most of the information that is available currently is from *in vitro* cell culture studies [3,17,25]. Some of the HDACi used in these studies include butyrate, tributyrin and TSA. In general, these HDACi arrest VSMC in G1-phase by downregulating cdk4, cdk6, cdk2, and upregulating Cip/Kip and INK families of cdk-inhibitors resulting in inhibition of Rb activation, thus failure to progress through S-phase [3,17,25]. However, these limited studies in VSMC have no information on effects on cyclin D1 except for our recent study [17]. Interestingly, our study reveals upregulation of cyclin D1 and D3 in butyrate treated VSMC implicating they may have cell-cycle-independent role in butyrate inhibited VSMC proliferation [17]. In the present study, effects of butyrate and TSA on VSMC is investigated to determine whether upregulation of cyclin D1 in VSMC is restricted to butyrate alone or common to other HDACi, and also to understand the mechanism and significance of cyclin D1upregulation in proliferation arrested VSMC.

## 2. Results and Discussion

### 2.1. Cytostatic and Cytotoxic Mode of Inhibition of VSMC Proliferation by Butyrate and TSA

**Figure 1:**
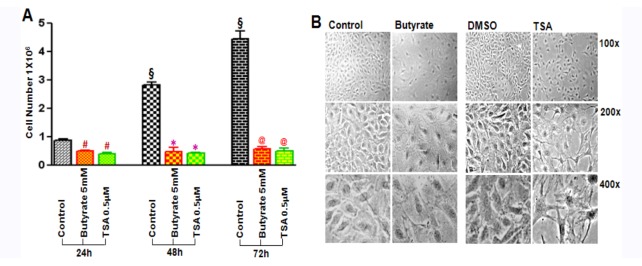
Effect of butyrate and TSA on VSMC proliferation and cellular morphology. Proliferating VSMC were treated with or without butyrate or TSA in complete medium as described in Experimental section for indicated periods of time. (**A**) VSMC proliferation was determined by cell counting; (**B**) Phase contrast images showing altered cellular morphology of VSMC treated with butyrate and TSA for 72 h at different magnifications. The values are expressed as the mean ± S.D. of three independent experiments. ^§^ *p* < 0.0001 *vs.* 24 h control; ^#^ *p* < 0.001 *vs.* 24 h control; * *p* < 0.0001 *vs.* 48 h control; ^@^ *p* < 0.0001 *vs.* 72 h control by ANOVA with the Bonferroni test.

Both butyrate and TSA inhibit VSMC proliferation but they arrest VSMC proliferation differently. Butyrate and TSA exhibit almost complete inhibition of VSMC proliferation at 5 mM and 0.5 μM concentration, respectively, with no cytotoxicity up to 48 h (Figure 1A), However, signs of cytotoxicity is observed when VSMC are treated longer than 48 h with 0.5 μM (Figure 1B), and higher than 0.5 μM TSA concentration (data not shown). Conversely, butyrate exhibits no toxicity at 5 mM concentration, consistent with our earlier studies, which revealed complete inhibition of VSMC proliferation with no cytotoxic effect from 5 to 8 mM [17,26]. Furthermore, VSMC treated with butyrate exhibit changes in cellular morphology from their typical elongated bipolar morphology to a round and spread-out phenotype and an increase in cell size. Treatment of VSMC with 0.5 μM TSA also causes subtle changes in morphology, with a slight increase in cellular size for the first 48 h, however, longer than 48 h treatment results in a more compressed cellular structure with long slender cellular extensions revealing signs of cytotoxicity (Figure 1B). Taken together it appears butyrate and TSA inhibit VSMC proliferation, but by cytostatic and cytotoxic effects, respectively. Moreover, these effects of butyrate and TSA on VSMC are consistent with their effects on other cell types. While butyrate arrests cell proliferation and stimulate morphological changes of variety of cell types, and in certain cell types the changes mirror the normal phenotype [13], TSA inhibits proliferation and causes cytotoxicity and apoptosis [27,28,29,30].

### 2.2. Disparate Effects of Butyrate and TSA on Cyclin D1 Expression in VSMC

G1-specific D-type cyclins, D1, D2 and D3 cyclins, are important cyclins that drive the cells through G1/S phase transition in response to mitogenic signals. Expression of D-type cyclins is the earliest event in the cell cycle progression, which is transiently induced by mitogenic signals, and their levels decline when mitogenic stimulus is withdrawn. Once induced, D-type cyclins associate with their catalytic partners, cdk4 and cdk6 and activate their kinase activity to drive phosphorylation and subsequent inactivation of Rb protein. This promotes release of E2F transcription factors to induce expression of target genes essential for entry into S phase. Interestingly, although D-type cyclin expression is induced mainly in response to mitogenic signals [31,32] our earlier studies reveal increased levels of cyclin D1 and D3 in VSMC that are proliferation arrested by butyrate unlike most other HDACi [17]. In order to determine whether this response of VSMC is unique to butyrate or common to HDACi, we compared the levels of cyclin D1 and D3 in VSMC treated with butyrate and TSA.

**Figure 2:**
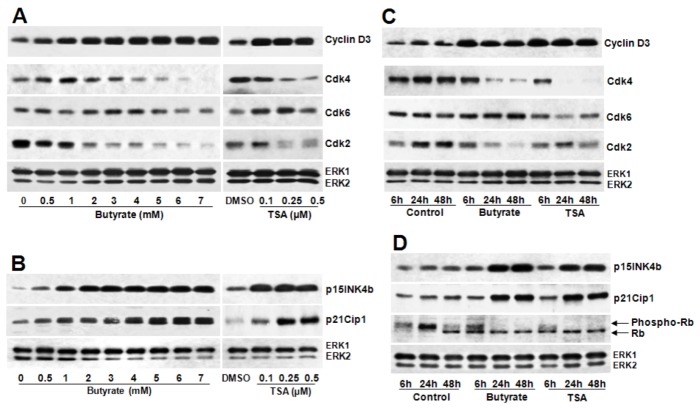
Comparison of butyrate and TSA effects on G1-specific cell cycle regulatory proteins. Proliferating VSMC were treated with different concentration of butyrate or TSA for 48 h (**A** and **B**) or treated with butyrate or TSA for indicated periods of time (**C** and **D**) to determine dose- and time-dependent effects of butyrate and TSA on G1-specific cell cycle proteins, respectively. At the end of treatment, cell lysates were prepared and processed for western analysis of cyclin D3, cdk4, cdk6, cdk2, p15INK4b, p21Cip1, Rb and phospho-Rb. Immunoblotting of ERK1/2 was performed with the same lysate to normalize the protein loading. Results shown are the representative of three independent experiments. The density of each band is measured and normalized to protein loading. The data obtained are analyzed and expressed as mean ± S.D. Expression of cyclin D3 (**A**), p21Cip1 and p15INK4b (**B**) are increased very significantly by I mM to 7 mM butyrate concentration *vs.* control (*p* < 0.001) and by 0.1 µM to 0.5 µM TSA concentration *vs.* DMSO (*p* < 0.001). Expression of cdk4 and cdk2 (**A**) are significantly decreased by 2 mM to 7 mM butyrate *vs.* control (*p* < 0.01) and by 0.25 µM to 0.5 µM TSA *vs.* DMSO (*p* < 0.01). Cdk6 is marginally affected by butyrate and TSA compared to their respective controls (**A**). Cyclin D3 (**C**), p21Cip1 and p15INK4b (**D**) are significantly increased both by 5 mM butyrate and 0.5 µM TSA *vs.* control (*p* < 0.001) at all experimental periods of time. Cdk4 and cdk2 are decreased by butyrate significantly after 6 h of treatment (*p* < 0.001) but no significant change in cdk6 *vs.* control (**C**). TSA causes significant reduction of cdk4 after 6 h (*p* < 0.001), cdk2 after 24 h (*p* < 0.05) and no significant effect on cdk6 (**C**) compared to control. Phosphorylation of Rb protein (**D**) is significantly inhibited both by butyrate and TSA after 6 h treatment compared to control (*p* < 0.001).

**Figure 3:**
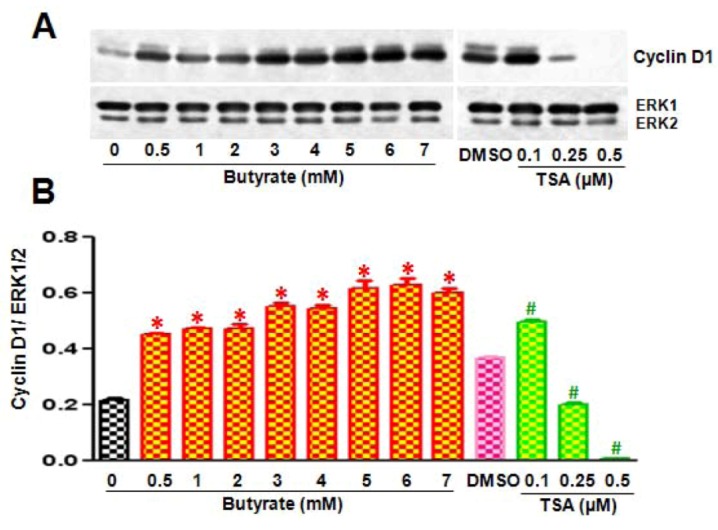
Disparate effects of butyrate and TSA on cyclin D1 expression in VSMC. Proliferating VSMC were treated with different concentrations of butyrate or TSA for 48 h and then cell lysates were prepared and processed for western analysis of cyclin D1. (**A**) Western blots were probed with cyclin D1-specific antibody and band intensities of cyclin D1 were determined and quantitated using a Molecular Imager FX Pro Plus MultiImager system and Quantity one software (Bio-Rad, CA, USA); (**B**) Band intensities are normalized to ERK1/2 as shown in bottom panel. Data are presented as mean ± S.D. of three independent experiments. * *p* < 0.0001 *vs.* no addition control (Con); ^#^ *p* < 0.0001 *vs.* DMSO vehicle control by ANOVA with the Bonferroni test.

Assessment of butyrate and TSA effects on G1-specific cell cycle regulatory proteins that regulate the phosphorylation/activation status of Rb protein, an essential event necessary for the cell to progress through G1 to S phase, by Western analysis reveals almost similar effects on most of the G1-specific cell cycle proteins, with the exception of cyclin D1. Temporal- (Figure 2A,B) and dose-dependent (Figure 2C,D) studies demonstrate increase in cyclin D3, downregulation of cdk4, cdk6, and cdk2 (Figure 2A,C), upregulation of cdk-inhibitors (cdkI) p21/Cip1 and p15INK4B (Figure 2B,D) and inhibition of Rb phosphorylation (Figure 2D). Conversely, both HDACi differently affect cyclin D1 in VSMC (Figure 3). While butyrate treatment of VSMC increases cyclin D1 levels in a concentration-dependent manner, treatment of VSMC with TSA prompts concentration-dependent reduction in cyclin D1 (Figure 3A). About a 3-fold increase in cyclin D1 protein is observed in VSMC treated with 3 to 7 mM butyrate at the end of 48 h of treatment. On the contrary, cyclin D1 levels are greatly reduced by TSA and at 0.5 µM, barely visible.

While the effects of butyrate and TSA on cdkI, p21Cip1, one of the genes that are universally upregulated in HDACi-induced proliferation arrest [11,16,17,33], and on cyclin-D-specific cdk4, cdk6, cyclin E-specific cdk2, p15INK4b and phosphorylation/activity state of Rb protein fits the portfolio for arrest of proliferation, their similar effects on cyclin D3 and differential effects on cyclin D1 is intriguing. However, butyrate has been shown to arrest proliferation of a wide range of cells and exhibit atypical effects on G1-specifc cyclin D1 and D3 in a cell type specific manner [34,35,36,37,38]. On the other hand, TSA, which has been shown to arrest proliferation and/or stimulate apoptosis of a variety of cells, causes downregulation of cyclin D1 [27,28,29,39,40,41], although in some cases TSA also increased cyclin D3 similar to butyrate [35]. Even though very limited information is available regarding the effects of butyrate [17,42] and TSA [3,25] on VSMC, particularly on D-type cyclins, upregulation of both cyclin D1 and D3 observed in VSMC in response to butyrate treatment appears to be unique to VSMC but significance of it is not clear. Recently it is recognized that besides functioning as cdk-dependent regulators of cell cycle, D-type cyclins exhibit crucial roles in the regulation of cdk-independent cellular processes depending on their levels of expression by modulating transcriptional activation. Perhaps butyrate induced D1 may have a role in the regulation of cdk-independent cellular processes such as increase in cellular size and altered cellular morphology.

#### 2.2.1. Influence of Butyrate and TSA on Phosphorylation State of Cyclin D1 in VSMC

The amount of cyclin D1 protein is stringently regulated via several mechanisms, including ubiquitin-26S proteasome-dependent protein degradation, protein intracellular localization and regulation of mRNA transcription. The stability of cyclin D1 plays a major role in the expression levels of cyclin D1. Threonine residue at position 286 (Thr-286) located near the carboxyl terminus of cyclin D1 plays a role in the stability [43,44]. Phosphorylation of this Thr-286 residue by GSK3, a serine/threonine kinase, is reported to trigger destability/proteasomal degradation of cyclin D1 resulting in decline in cyclin D1 levels.

**Figure 4:**
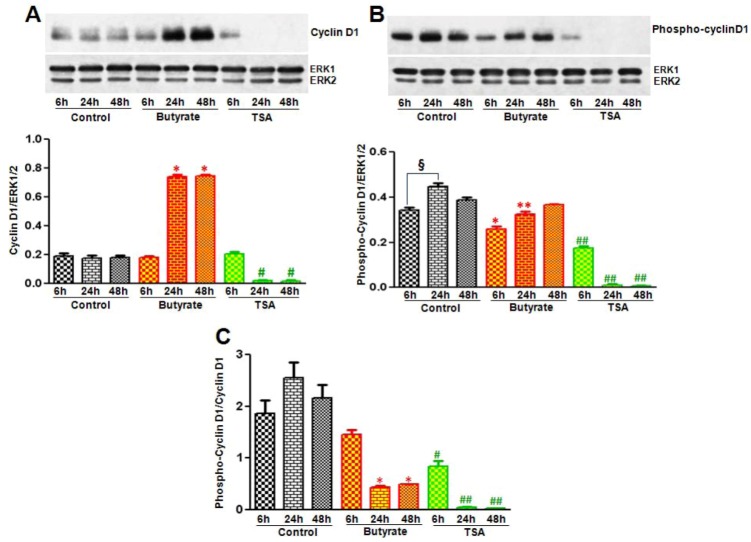
Assessment of butyrate and TSA effects on cyclin D1 phosphorylation state. VSMC were treated with butyrate or TSA for required periods of time. At the end of treatment, cell lysates were prepared and processed to assess by western analysis total cyclin D1 and phosphorylated cyclin D1. Respective data are presented as mean ± S.D. of three independent experiments. (**A**) Total cyclin D1 levels were determined by using cyclin D1-specific antibody. The band intensities are normalized to ERK1/2 (bottom panel). * *p* < 0.0001 *vs.* no addition control; ^#^ *p* < 0.001 *vs.* DMSO vehicle control by ANOVA with the Bonferroni test; (**B**) Phospho-cyclin D1 levels were evaluated by using antibody specific to Thr-286-phosphorylated cyclin D1. Band intensities were normalized to ERK1/2 (bottom panel). ^§^ *p* < 0.01 *vs.* 6 h control; * *p* < 0.01 *vs.* 6 h control; ** *p* < 0.001 *vs.* 24 h control; ^##^ *p* < 0.0001 *vs.* respective controls; (**C**) Ratio of phospho-cyclin D1 to total cyclin D1 were determined by using the values presented in Figure 4A,B and displayed in the bottom panel. * *p* < 0.001 *vs.* respective controls; ^#^ *p* < 0.001 *vs.* 6 h control; ^##^ *p* < 0.0001 *vs.* respective controls.

To assess whether changes in stability/degradation contributes to the differences in the cyclin D1 levels in VSMC treated with butyrate and TSA, phosphorylated cyclin D1 levels are determined by western analysis with an antibody specific to phosphorylated Thr-286 cyclin D1. Treatment of VSMC with butyrate not only causes time-dependent increase in cyclin D1 levels (Figure 4A) but also reveals significant reduction in phospho-cyclin D1 levels (Figure 4B) and ratio of phospho-cyclin D1/total cyclin D1 (Figure 4C). On the contrary, while VSMC exposed to TSA for 6 h exhibits detectable levels of cyclin D1, it is reduced to undetectable levels after 6 h of treatment (Figure 4A). Similarly, phosphorylated cyclin D1 is detectable only in VSMC treated for 6 h (Figure 4B) and the ratio of phospho-cyclin D1/total cyclin D1 is close to about one (Figure 4C). All in all, it appears significant protein stability combined with upregulated synthesis is responsible for augmented cyclin D1 levels in butyrate treated VSMC. Whereas decreased protein stability and no change in synthesis seem to cause reduction in cyclin D1 levels in TSA treated VSMC.

#### 2.2.2. Subcellular Localization of Cyclin D1 Protein in VSMC Treated with HDACi

**Figure 5:**
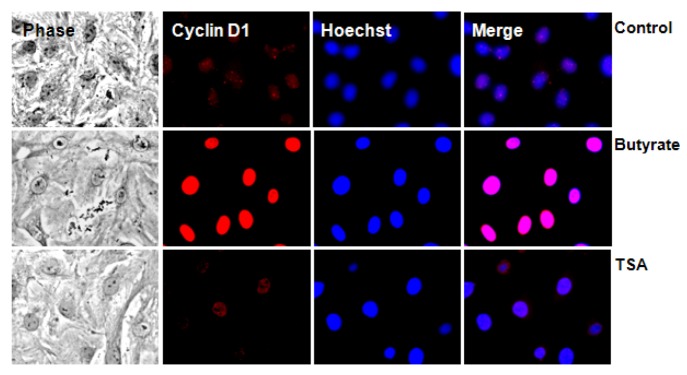
Intracellular localization of cyclin D1 in butyrate and TSA treated VSMC. VSMC treated with 5 mM butyrate or 0.5 µM TSA for 24 h were immunostained with cyclin D1 antibody followed by nuclear staining with Hoechst. Images of stained VSMC were captured using a Nikon fluorescence microscope with a CCD camera (400× magnification).

The subcellular distribution of D-type cyclins is regulated by cell cycle-dependent events. Cyclin D1 accumulates in the nuclei of cells during G1 phase, but once cells enter S phase and DNA replication begins, cyclin D1 gets phosphorylated by GSK3, which causes increased association of cyclin D1 with a nuclear exportin, CRM 1 [45]. This facilitates nuclear exit and transport to cytoplasm where cyclin D1 undergoes subsequent degradation via 26S proteasome [44]. Since butyrate increases cyclin D1 levels but exhibits limited cyclin D1 phosphorylation in VSMC, substantial amounts of butyrate-induced cyclin D1 is expected to be sequestered in nucleus. This possibility is explored by immunostaining of VSMC for cyclin D1, which not only confirms differential effects of butyrate and TSA on cyclin D1 levels but also reveals butyrate-induced cyclin D1 is solely localized in nuclear region, which is barely visible in VSMC treated with TSA (Figure 5).

Considering the role that GSK3 plays in cell cycle regulation through the control of cyclin D1 levels, it appears the differences observed in the cyclin D1 phosphorylation state in VSMC treated with HDACi suggests that ineffective phosphorylation of cyclin D1 by GSK3 (Figure 4) is preventing nuclear exit of cyclin D1 in butyrate treated VSMC. Consequently, it results in cyclin D1 accumulation in nucleus (Figure 5). GSK3, a ubiquitously expressed cytoplasmic serine/threonine protein kinase has been shown to have crucial role in cell cycle regulation through its control of cyclin D1 proteolysis by direct phosphorylation of cyclin D1 [44]. Although identified initially as the kinase that phosphorylates glycogen synthase to inhibit glycogen synthesis, GSK3 is now recognized to play central role in a variety of biological functions, including development, metabolism, gene transcription, protein translation, cytoskeletal organization/integrity, cell cycle regulation, and apoptosis [44,46]. Two isoforms of GSK3, GSK3α and GSK3β have been identified, which exhibit a high degree of amino acid homology and appear to function in a similar manner in most cell systems, however, the GSK3β isoform appears to be the predominant isoform in adult cells [46]. The activity of GSK3 is regulated by multiple mechanisms. A major mechanism for cell cycle regulation is the inhibition of GSK3 activity by serine-phosphorylation. Phosphorylation at serine 21 and serine 9 of GSK3α and GSK3β isoforms of GSK3, respectively, inhibits GSK3 activity. This inhibitory phosphorylation is induced by upstream protein kinases of different signaling pathways including PI3Kinase/Akt pathway [44,46]. It is possible that the upstream signaling pathways may be inhibiting the activity of GSK3 in butyrate treated VSMC by promoting phosphorylation of GSK3, which causes ineffective phosphorylation of cyclin D1 by GSK3. As a result, cyclin D1 accumulates in nucleus (Figure 5).

#### 2.2.3. Cyclin D1 Levels Are Not Regulated by GSK3 in VSMC Treated with Butyrate and TSA

**Figure 6:**
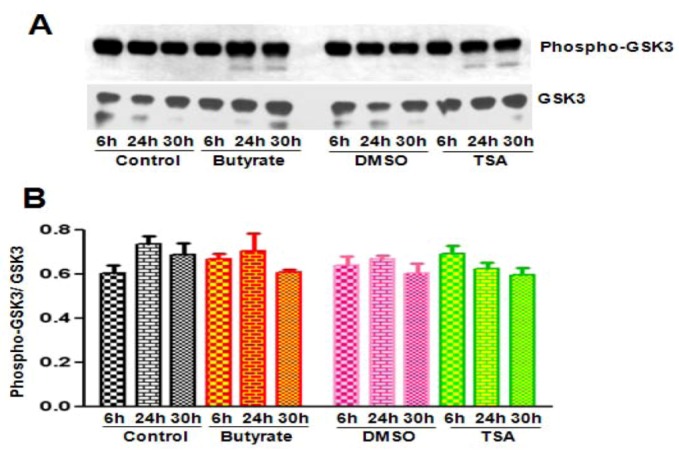
GSK3 activity in VSMC treated with butyrate and TSA. Proliferating VSMC were treated with butyrate or TSA for indicated periods of time. Cell lysates were prepared and subjected to western analysis for phosphorylated GSK3α/β (Ser21/Ser9), together with total GSK3α/β using appropriate antibodies (**A**) The results were quantitated and ratio of phosphorylated to non-phosphorylated total GSK3 was determined; (**B**) Data are presented as mean ± S.D. from three independent experiments. Analysis of the data indicates no significant difference in GSK3 activity between untreated and treated groups.

Because butyrate and TSA exhibit different effects on the levels of total cyclin D1 and Thr-286 phosphorylated cyclin D1 in VSMC, the possibility that the HDACi may differently alter activity of GSK3, the enzyme that catalyzes phosphorylation of Thr-286 residue of cyclin D1, is investigated. GSK3 activity state is analyzed by Western analysis with phospho-GSK3α/β (Ser21/Ser9) antibody specific to inhibitory phosphorylation of GSK3α (Ser21)/ GSK3β (Ser9) (Figure 6A). Total GSK3 is also determined similarly with an antibody specific to GSK3α/β to measure the ratio of the phospho-GSK3/total GSK3 (Figure 6B). Although low levels of GSK3β expression is detected in VSMC, surprisingly both untreated and HDACi treated VSMC exhibit almost similar levels of inactive phosphorylated GSK3α/β (Ser21/Ser9) and total GSK3α/β. No significant differences observed in both the levels of inhibitory phosphorylation of GSK3α/β and in the ratio of phospho-GSK3α/β (Ser21/Ser9) /total GSK3α/β suggesting GSK3-independent mechanism may be involved in the differential regulation of cyclin D1 in VSMC treated with HDACi [43,44]. Moreover, studies of Yong *et al.* [47] have reported that GSK3 has limited role in cell-cycle-regulated cyclin D1 levels. It is also recognized that besides Thr-286, the target for GSK3-mediated cyclin D1 phosphorylation/degradation, another threonine motif, Thr-288, is reported to have an important role in cell cycle related phosphorylation/degradation of cyclin D1 [48]. A member of the DYRK family, dual-specificity tyrosine-phosphorylation regulated kinase 1B (DYRK1B) is shown to phosphorylate cyclin D1 on Thr-288 resulting in its degradation. At this moment, we are not sure, whether this pathway of cyclin D1 regulation participates in the differential effects of butyrate and TSA on cyclin D1 in VSMC.

### 2.3. Differential Effects of HDACi on Cyclin D1 Transcription

**Figure 7:**
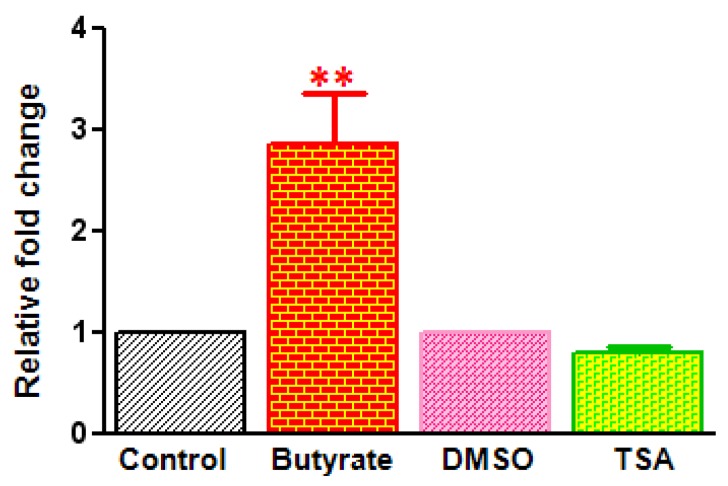
Cyclin D1 gene expression in butyrate and TSA treated VSMC. VSMC were treated with 5 mM butyrate or 0.5 µM TSA for 48 h and total RNA was isolated and subjected to real-time RT-PCR to quantitate cyclin D1 gene transcription. 18S ribosomal RNA was used as a normalizing control. Data are presented as mean ± S.D. from three independent experiments. ** *p* < 0.001 compared to control VSMC.

To determine whether the differential effects of butyrate and TSA on cyclin D1 levels are due to altered cyclin D1 gene expression, effects of these HDACi on cyclin D1 mRNA transcription are followed by Real-time RT-PCR (Figure 7). The data clearly indicates upregulation of cyclin D1 expression in butyrate treated VSMC. Cyclin D1 expression is increased about 2.5-fold in VSMC treated with butyrate. Conversely, cyclin D1 transcription is decreased slightly in TSA treated VSMC. In general, it appears that increase in cyclin D1 levels in butyrate treated VSMC is associated with increase in synthesis and decrease in degradation. Whereas reduced cyclin D1 levels in TSA treated VSMC is mainly ascribed to protein degradation, however, the slight reduction in cyclin D1 transcript levels also contributes to reduced cyclin D1 levels.

### 2.4. Portraits of Butyrate and TSA-Induced Site-Specific Posttranslational Modifications of Histone H3 in VSMC

**Figure 8:**
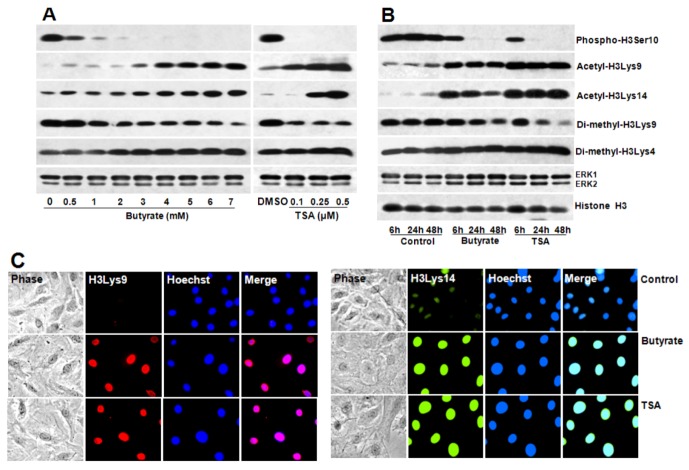
Concentration and time-dependent changes in site-specific modifications of histone H3 in butyrate and TSA treated VSMC. Proliferating VSMC were treated with different concentration of butyrate or TSA for 48 h (**A**) or treated with 5 mM butyrate or 0.5 µM TSA for indicated periods of time (**B**); to determine dose- and time-dependent effects of butyrate and TSA on indicated site-specific modifications of histone H3, respectively. At the conclusion of the treatment, VSMC were processed for western analysis using appropriate antibodies to assess the effects of butyrate and TSA on histone H3 modifications. ERK1/2 was immunoblotted and used as loading control. The results shown are representative of three independent experiments. The density of each band is measured and normalized to protein loading. The data obtained are analyzed and expressed as mean ± S.D. Phospho-H3Serine10, acetyl-H3Lys9, acetyl-H3Lys14, di-methyl-H3Lys9 and di-methyl-H3Lys4 modifications stimulated by butyrate *vs.* control are significant to very significant (*p* < 0.01 to *p* < 0.001) depending on the concentrations of butyrate except for di-methyl-H3Lys4 modification, which is not significant at 0.5 and 1 mM butyrate concentration (**A**). Changes induced by TSA *vs.* DMSO are significant to very significant (*p* < 0.01 to *p* < 0.001) with the exception of acetyl-H3Lys14 and di-methyl-H3Lys4 at 0.1 µM TSA (**A**). Time-dependent changes in levels of phospho-H3Serine10, acetyl-H3Lys9, acetyl-H3Lys14, di-methyl-H3Lys9 and di-methyl-H3Lys4 histone H3 modifications are significant to very significant (*p* < 0.05 to *p* < 0.001) both in butyrate and TSA treated VSMC compared to untreated control at all periods of time with the exception of di-methyl-H3Lys9 at 6 h (**B**); (**C**) Effect of butyrate and TSA on acetylation of H3Lys9, and H3Lys14 was also determined by intracellular immunofluorescence staining of H3Lys9, and H3Lys14 followed by nuclear staining with Hoechst. VSMC treated with butyrate or TSA for 24 h were immunostained with respective antibodies and images of immunostained VSMC were captured using Nikon fluorescence microscope with a CCD camera (400× magnification).

To determine whether differential expression of cyclin D1 in butyrate and TSA arrested VSMC proliferation is linked to change in chromatin structure, changes in site-specific posttranscriptional modifications (PTMs) of histone H3, specifically, H3Ser10 phosphorylation, H3Lys9 and H3Lys14 acetylation, and H3Lys9 and H3Lys4 di-methylation are assessed (Figure 8). Results of this study indicate that both butyrate and TSA treated VSMC display more or less similar profiles of different site-specific PTMs of histone H3. Butyrate and TSA treated VSMC reveal time- and dose-dependent: inhibition of H3Ser10 phosphorylation associated with cell cycle/mitosis, induction of H3Lys9 and H3Lys14 acetylation linked to transcriptional activation, reduction of H3Lys9 di-methylation and stimulation H3Lys4 di-methylation related to transcriptional suppression and activation, respectively (Figure 8A,B) [17,49,50,51,52,53].

Furthermore, induction of H3Lys9 and H3Lys14 acetylation by butyrate and TSA is confirmed by immunostaining of VSMC with antibodies that are specific to acetylated histone H3Lys9 and H3Lys14 (Figure 8C). These observations indicate butyrate and TSA cause similar global PTMs of histone H3 implicating these PTMs alter chromatin structure similarly. Although these global changes do not provide information regarding the epigenetic changes associated with differential expression of cyclin D1 by butyrate and TSA in VSMC, analysis of histone H3 PTMs at cyclin D1 promoter region may provide clues to the influence of histone epigenetic modifications in the differential expression of cyclin D1 in response to HDACi.

## 3. Experimental

### 3.1. Materials

Butyrate was obtained from Sigma-Aldrich (St. Louis, MO, USA). Trichostatin A and antibodies to cyclin D1, phospho-cyclin D1, cyclin D3, p15INK4B, extracellular signal-regulated kinase 1 and 2 (ERK1/2), glycogen synthase kinase-3α/β (GSK-3α/β), phospho-GSK-3α/β, histone H3, phospho-histone H3Serine10 (phospho-H3Ser10), acetyl-histone H3Lysine9 (acetyl-H3Lys9), di-methyl-histone H3Lysine9 (di-methyl-H3Lys9), di-methyl-histone H3Lysine4 (di-methyl-H3Lys4), phospho-Rb-Serine807/811, (pRbSer807/811), and horse radish peroxidase (HRP)-conjugated second antibodies were obtained from Cell Signaling Technology (Boston, MA, USA). Antibodies to acetyl-histone H3Lysine14 (acetyl-H3Lys14) was from Abcam (Cambridge, MA, USA). Anti-mouse Alexa Fluor 488, anti-rabbit Alexa Fluor 546, and Hoechst were from Molecular Probes (Life Technologies, Grand Island, NY, USA). Chemiluminescence luminol reagent and antibodies to p21Cip1, cdk2, cdk4 and cdk6 were obtained from Santa Cruz Biotechnology (Santa Cruz, CA, USA). Antibody to Rb protein was purchased from BD Biosciences (San Jose, CA, USA). The micro BCA protein assay kit was from Pierce (Rockford, IL, USA). RNeasy Plus Mini kit from QIAGEN (Valencia, CA, USA). RT^2^ EZ First Strand kit, RT^2^ SYBR Green qPCR Mastermix, and rat cyclin D1 (PPR06517C) and 18S ribosomal RNA (PPR57734E) primer sets were from SABiosciences (Frederick, MD, USA).

### 3.2. Cell Culture and Treatments

Rat VSMC isolated from thoracic aortas [54,55] were cultured in Dulbecco’s modified Eagle’s medium supplemented with 10% fetal bovine serum, 100 U/mL penicillin and 100 μg/mL streptomycin (complete medium) at 37 °C in a humidified atmosphere of 95% air and 5% CO_2_. For all experiments, VSMC were seeded at a ratio of 1:6. One day after splitting, actively growing cells were treated with 5 mM butyrate or 0.5 μM TSA unless otherwise specified for required lengths of time [17,26]. Control cells were treated with an equivalent amount of phosphate-buffered saline (PBS) or DMSO, the vehicles used for dissolving butyrate and TSA, respectively. VSMC Culture medium was replaced every other day with fresh medium containing butyrate or TSA. VSMC of third to fifteenth passages were used for all studies. All experiments were repeated at least three times unless otherwise mentioned.

### 3.3. Measurement of Cell Proliferation

After required periods of treatment, cells were washed three times with sterile PBS and trypsinized with trypsin-EDTA. Cell numbers were counted by trypan blue exclusion method as described previously [17,26].

### 3.4. Western Analysis

At the end of experiment, cultures were washed with ice-cold PBS and whole cell lysates were prepared as described previously [17]. Equal amounts of denatured protein samples were fractionated on: 7.5%, 10% and 12% SDS-polyacrylamide gels to immunoblot Rb and phospho-Rb; cyclin D1, cyclin D3, phospho-cyclin D1, GSK-3α/β, phospho-GSK-3α/β, ERK1/2, p21Cip1, cdk2, cdk4 and cdk6; and p15INK4B, unmodified and modified histone H3, respectively. Fractionated proteins were transferred to PVDF membrane and processed for immunoblotting with appropriate antibodies and western blot luminol reagent from Santa Cruz Biotechnology was used for immunodetection. For quantitative measurements, a Molecular Imager FX Pro Plus MultiImager System and Quantity One software from Bio-Rad (Hercules, CA, USA) were used. Immunoblotting of ERK1/2 was performed with the same lysates to normalize protein loading unless otherwise mentioned.

### 3.5. Immunofluorescence

VSMCs were fixed in cold methanol and immunostained as described previously [17,26]. Briefly, fixed cells were blocked with 10% heat inactivated horse serum (HS) in PBS for 1 h at room temperature. Blocked cells were incubated with appropriate antibodies in 1.5% HS for 1 to 3 h followed by three washes with PBS, each for 10 min. Similar washing protocol is used for downstream procedures. Cultures were then incubated with appropriate Alexa Fluor second antibody conjugates in 1.5% HS for 1 h. Following washing, cultures were incubated with 1 μg/mL Hoechst in 1.5% HS for 30 min. After washing with PBS cultures were subjected to fluorescence microscopy using a Nikon fluorescence microscope.

### 3.6. Real-Time Quantitative RT-PCR

After 24 h of plating, proliferating VSMC were treated with no addition, 5 mM butyrate or 0.5 μM TSA. After 48 h, total RNA was isolated from these experimental VSMC cultures using RNeasy Plus Mini kit from QIAGEN, which is designed to efficiently purify RNA by integrating a step that allows selective removal of genomic DNA. About 4 μg of purified RNA samples were reverse transcribed with RT^2^ EZ First Strand kit from SABiosciences to obtain cDNAs. Appropriate amounts of each cDNA were amplified using RT^2^ SYBR Green qPCR Mastermix from SABiosciences on a Bio-Rad iCycler Real-Time PCR system. Rat cyclin D1 (PPR06517C)-specific primer set was used together with primer set for rat 18S ribosomal RNA (PPR57734E) as the house-keeping normalization control from SABiosciences. Each amplification reactions were performed in triplicates by following a two-step cycling program prescribed for the RT^2^ SYBR Green qPCR Mastermix by the manufacturer (SABiosciences): one cycle of 95 °C for 10 min, and 40 cycles of 95 °C for 15 s and 60 °C for 1 min. Negative PCR controls including no reverse transcription, and omission of cDNA or primers were used to validate each batch of template before use. The individual data were normalized to 18S rRNA value and ΔΔC_T_ values were determined to calculate the fold-change in cyclin D1 expression.

### 3.7. Data Analysis

Data is expressed as mean ± SD. Statistical significance of the data was evaluated using one-way analyses of variance (ANOVA) with Bonferroni multiple comparison test. Statistically significant difference between data sets was determined at *p* < 0.01 to < 0.0001. Statistical analysis was performed using GraphPad Prism version 5 software from Sigma-Aldrich (St. Louis, MO, USA).

## 4. Conclusions

In the present study, although both butyrate and TSA exhibit increase in cyclin D3, selective induction of cyclin D1via increased transcriptional activity by butyrate appears to be unique to VSMC, although its significance is not clear. Additionally, butyrate causes striking changes in cell size and morphology [13,17,56], in addition to arresting VSMC proliferation. Considering that D-type cyclin expression is induced mainly in response to mitogenic signals, upregulated cyclin D1 expression in butyrate arrested VSMC proliferation may have cdk-independent roles, besides its cdk-dependent role in the inactivation of Rb protein, potentially in altered cellular morphology as evidenced in the NGF-mediated stimulation of neurite outgrowth in PC12 cells [57,58]. Evidence indicates that D-type cyclins exhibit non-redundant crucial roles in the regulation of cdk-independent cellular processes depending on their levels of expression, the specific cell type, the cell context, and other factors by regulating transcriptional activation [57]. Cyclin D1 has been implicated in the regulation of cellular metabolism, cellular migration, cellular survival, angiogenesis, inhibition of myogenic and adipogenic differentiation and stimulation of neurite outgrowth [57,58]. The mechanisms by which D1 cyclin regulate such distinct functions and exhibit disparate effects are not clear. However, studies have recognized functional interaction between cyclin D1 with diverse transcription factors and transcriptional regulators including HATs and HDACs [57,59]. In fact by forming physical associations with more than 30 different distinct transcription factors or transcriptional regulators, cyclin D1 has been suggested to regulate their activity, thus influencing cellular effects [57]. It is possible that the differences in cyclin D1 expression observed in VSMC in response to butyrate and TSA treatment in the present study may be linked to transcriptional regulation of genes associated with changes in cellular morphology/cellular effect-induced by these HDACi. Butyrate causes cytostatic effect with change in cell shape and increase in size, which may involve cyclin D1-mediated cdk-independent mechanism unlike TSA that appears to trigger cytotoxicity.

The difference in cellular effects of butyrate and TSA implicate butyrate may be a better choice for therapeutic application of cell cycle inhibition, particularly in vascular proliferative diseases such as arterial restenosis, in-stent restenosis, transplant vasculopothy and vein bypass graft failure where VSMC proliferation is the primary pathological mechanism. Moreover, butyrate is a cytoprotective agent and a bioactive component of dietary fiber, which is suggested to mediate the health benefits of dietary fiber in chronic diseases including cardiovascular diseases [1,3,4,13,14,15]. Different approaches have been tried for arresting VSMC proliferation by targeting cell cycle, which include brachytherapy or use of radiation, gene therapy, and pharmacotherapy [1,4]. These approaches inhibit proliferation by cytostatic or cytotoxic mechanism. However, cytostatic mechanism of cell cycle arrest is desired over cytotoxic mechanism to avoid unintended damage to the vessel wall due to cytotoxic treatment.
